# Supplementary material for: A quantitative look on northwestern Tethyan foraminiferal assemblages, Campanian Nierental Formation, Austria
Source: PeerJ. 2016 Mar 8;4:e1757. doi: 10.7717/peerj.1757 (PMC4793319; doi:10.7717/peerj.1757)
Supplement: Appendix S1 — Appendix Table 1. Depth range and distribution of benthic foraminiferal taxa at Postalm. Depth ranges are inferred from the literature and were reconstructed using Nyong and Olson (1977) with depth ranges for Campanian-Maastrichtian benthic foraminifera along the Atlantic coast, as well as the bathymetric ranges of benthic foraminifera of Sliter & Baker (1972), Speijer & Van der Zwaan (1996), Kaminski & Gradstein (2005), Valchev (2006) as well as Holbourn, Henderson & MacLeod (2013). [file peerj-04-1757-s001.doc]

|  | estimated depth range | |  |  |  |  |  |  |  |  |  |  |  |  |  |  |  |  |  |  |  |  |  |  |  |  |
| --- | --- | --- | --- | --- | --- | --- | --- | --- | --- | --- | --- | --- | --- | --- | --- | --- | --- | --- | --- | --- | --- | --- | --- | --- | --- | --- |
|  | min | max | POST_07/34 | POST_07/35 | POST_07/36 | POST_07/37 | POST_07/38 | POST_07/39 | POST_07/40 | POST_07/41 | POST_07/42 | POST_07/43 | POST_07/44 | POST_08/01 | POST_08/02 | POST_08/03 | POST_08/04 | POST_08/05b | POST_08/05a | POST_08/06 | POST_08/07 | POST_08/08 | POST_08/09 | POST_08/10 | POST_08/11 | POST_08/12 |
| *Allomorphina cretacea* | 500 | 3,500 |  |  |  |  |  |  |  |  |  |  |  |  |  |  |  |  |  |  | **X** |  |  |  |  |  |
| *Ammodiscus cretaceus* | 100 | 3,000 |  |  |  |  |  |  |  |  |  |  | **X** |  |  |  |  |  |  |  |  |  |  |  |  |  |
| *Ammo*sp*haeroidina pseudopauciloculata* | 150 | 4,000 | **X** |  |  |  | **X** | **X** |  |  |  |  |  | **X** |  |  | **X** |  |  |  |  |  |  |  |  |  |
| *Anomalinoides larseni* | 1,500 | 4,000 |  |  |  |  |  |  |  |  |  |  |  |  |  |  |  |  |  |  | **X** |  |  |  |  |  |
| *Bathysiphon* sp*.* | 500 | 2,000 |  |  |  |  | **X** |  |  |  |  |  |  |  |  |  |  |  |  |  | **X** |  | **X** |  |  |  |
| *Bolivinoides clavatus* | 500 | 3,000 |  |  | **X** |  |  |  |  |  | **X** |  |  |  |  |  |  |  |  |  |  |  |  |  |  |  |
| *Bulimina obtusa* | 600 | 2,000 | **X** |  |  |  |  |  |  |  |  | **X** |  |  |  |  |  |  |  | **X** |  | **X** |  | **X** |  |  |
| *Caudammina excelsa* | 500 | 3125 |  |  |  |  |  |  |  | **X** |  | **X** |  |  |  | **X** |  |  |  | **X** |  |  |  |  | **X** |  |
| tubular agglutinates (cfAmmobaculites) | 150 | 4,000 |  |  | **X** | **X** | **X** |  |  |  |  | **X** |  |  |  |  |  |  |  |  |  |  |  |  | **X** |  |
| *Clavulina amorpha* | 200 | 2,500 | **X** |  |  |  |  |  |  | **X** |  |  |  | **X** |  |  | **X** |  | **X** |  |  | **X** |  |  |  |  |
| *Clavulina trilatera* | 200 | 2,000 |  | **X** | **X** | **X** | **X** | **X** | **X** |  |  | **X** |  |  |  |  |  |  |  |  |  |  |  |  |  |  |
| *Conglophragmium irregularis* | 200 | 4,000 |  |  |  | **X** |  |  |  |  |  |  |  |  |  |  |  |  |  |  |  |  |  |  |  |  |
| *Conorboides* sp*.* | 1 | 4,000 |  |  |  |  | **X** |  |  |  |  |  |  |  |  |  |  | **X** |  |  |  | **X** |  |  |  |  |
| *Cribrostomoides* spp*.* | 400 | 3,000 | **X** |  | **X** | **X** | **X** | **X** | **X** | **X** | **X** | **X** | **X** | **X** | **X** |  | **X** | **X** | **X** |  |  |  | **X** | **X** |  |  |
| *Dentalina annulata* | 50 | 2,500 |  |  | **X** |  |  |  |  |  |  |  |  |  |  |  |  |  |  |  |  |  | **X** |  |  |  |
| *Dentalina catenula* | 50 | 2,500 |  | **X** |  |  |  |  |  |  |  |  |  |  |  | **X** |  |  | **X** |  | **X** |  |  |  | **X** |  |
| *Dentalina* sp*.* | 50 | 2,500 | **X** |  | **X** | **X** | **X** | **X** |  |  | **X** | **X** |  |  | **X** |  |  |  |  | **X** |  | **X** |  |  |  |  |
| *Dentalina* sp*.*1(thick wall) | 50 | 2,500 |  |  |  |  |  | **X** |  |  |  |  |  |  |  |  | **X** |  |  |  |  |  |  |  |  |  |
| *Dentalina* sp*.*2 *pustulae (?)* | 50 | 2,500 |  |  |  |  |  | **X** | **X** |  |  |  |  |  |  | **X** |  |  |  |  |  |  |  |  |  |  |
| *Dentalina* sp.3 (aculeate term. chmb.) | 50 | 2,500 |  |  |  |  |  |  |  |  |  |  |  |  |  |  | **X** |  |  |  |  |  |  |  |  |  |
| *Dentalina* sp*.*4 (aculeate term. chamber) | 50 | 2,500 |  | **X** |  |  |  |  |  |  |  |  |  |  |  |  |  |  |  |  |  |  |  |  |  |  |
| *Dorothia conula* | 200 | 4,000 |  |  | **X** |  |  | **X** | **X** | **X** |  | **X** |  |  | **X** |  |  |  |  |  |  | **X** | **X** | **X** | **X** |  |
| *Dorothia pupoides* | 200 | 4,000 |  |  |  |  |  |  |  |  |  |  |  | **X** |  |  |  |  |  |  |  |  | **X** |  |  |  |
| *Dorothia*sp*.* | 200 | 4,000 |  |  |  |  | **X** | **X** |  |  |  |  |  | **X** |  |  |  |  |  |  |  |  |  |  |  |  |
| *Eponides beisseli* | 150 | 2,700 | **X** | **X** | **X** |  | **X** | **X** | **X** | **X** | **X** |  | **X** | **X** | **X** | **X** | **X** | **X** | **X** |  | **X** | **X** | **X** | **X** | **X** |  |
| Eponides sp.1 | 150 | 2,700 |  |  |  |  |  |  |  |  |  |  |  |  |  |  |  |  |  |  |  |  |  |  | **X** |  |
| *Globorotalites multiseptus* | 150 | 2,700 |  | **X** | **X** | **X** | **X** |  | **X** | **X** | **X** | **X** |  |  |  |  |  |  |  |  |  |  |  |  | **X** | **X** |
| *Gaudryina laevigata* | 200 | 2,000 |  |  |  |  |  |  |  |  |  |  |  | **X** |  |  |  |  |  |  |  |  | **X** |  | **X** | **X** |
| *Gaudryina pyramidata* | 200 | 2,000 | **X** | **X** |  | **X** |  |  | **X** |  |  | **X** | **X** | **X** | **X** | **X** | **X** | **X** |  |  | **X** | **X** | **X** |  |  |  |
| *Gaudryina rugosa* | 200 | 2,000 |  |  |  |  |  | **X** |  | **X** |  |  |  |  |  |  |  |  |  |  | **X** | **X** |  |  |  |  |
| *Gaudryina* sp*.* | 200 | 2,000 | **X** |  | **X** | **X** |  | **X** |  |  |  |  |  |  | **X** |  | **X** |  | **X** |  | **X** |  |  | **X** |  |  |
| *Gaudryina* sp.2 | 200 | 2,000 | **X** |  |  |  |  |  |  | **X** |  |  |  |  |  |  |  |  |  |  | **X** | **X** | **X** |  |  |  |
| *Guttulina communis* | 200 | 1,600 |  |  |  |  |  |  |  |  |  |  |  |  |  |  |  |  |  |  |  | **X** |  |  |  |  |
| *Guttulina* sp*.* | 200 | 1,600 | **X** |  |  |  | **X** | **X** |  |  |  | **X** |  |  |  | **X** |  |  |  |  |  | **X** |  |  |  |  |
| *Guttulina trigonula* | 200 | 1,600 |  |  |  |  |  |  |  |  |  |  |  |  |  |  |  |  |  |  |  |  |  |  |  |  |
| *Gyroidinews nitidus* | 200 | 2,700 |  |  |  | **X** |  |  |  |  |  |  | **X** |  |  |  |  | **X** |  | **X** | **X** | **X** |  | **X** |  |  |
| *Gyroidinoides lenticulus* | 200 | 2,700 |  |  |  |  |  |  |  |  |  |  |  |  |  |  |  | **X** |  | **X** |  |  |  |  |  |  |
| *Hyperammina* sp*.* | 100 | 4,000 |  |  |  |  |  |  |  |  |  |  | **X** |  |  |  | **X** |  |  |  | **X** |  | **X** |  |  | **X** |
| *Lagena apiculata* | 100 | 4,000 | **X** | **X** |  |  |  | **X** |  | **X** |  | **X** | **X** |  |  |  |  |  |  |  |  |  |  | **X** |  |  |
| *Lagena emaciata* | 100 | 4,000 |  |  |  |  |  |  |  |  |  |  |  |  |  |  |  |  |  |  |  |  | **X** | **X** | **X** |  |
| *Lagena* sp*.* | 100 | 4,000 | **X** |  |  |  | **X** | **X** |  | **X** |  | **X** |  |  |  |  |  |  |  |  |  |  |  |  |  |  |
| *Lenticulina* sp*.* | 50 | 3,500 |  | **X** | **X** | **X** |  | **X** |  |  | **X** |  |  |  |  | **X** |  |  |  |  |  |  |  |  | **X** |  |
| *Lenticulina* sp*.*1 | 50 | 3,500 |  |  |  |  |  |  |  |  |  | **X** |  |  |  | **X** |  |  |  |  |  |  |  |  |  |  |
| *Lenticulina subangulata* | 50 | 3,500 |  |  |  |  |  |  |  |  |  |  |  |  |  |  |  |  |  |  |  |  | **X** | **X** |  | **X** |
| *Lenticulina velascoensis* | 50 | 3,500 | **X** |  |  | **X** |  |  |  |  |  |  |  |  |  |  |  |  |  |  |  |  |  |  |  |  |
| *lenticulinites rotulata* | 50 | 3,500 |  | **X** | **X** |  |  |  |  |  |  |  |  | **X** | **X** |  | **X** |  |  |  |  |  |  |  |  |  |
| *Marginullina* sp | 50 | 4,000 | **X** |  |  |  |  |  |  |  |  |  |  | **X** |  |  | **X** |  |  |  | **X** | **X** |  |  |  |  |
| *Marssonella oxycona* | 200 | 4,000 |  |  |  | **X** |  | **X** |  |  | **X** | **X** |  |  |  |  |  |  |  |  |  |  | **X** |  | **X** |  |
| *Marssonella* sp*.* | 200 | 4,000 |  |  |  | **X** | **X** |  |  |  |  |  |  |  | **X** | **X** |  |  |  | **X** |  | **X** |  |  |  |  |
| *Marssonella trochus* | 200 | 4,000 |  |  |  |  |  |  |  |  |  |  |  |  |  |  |  |  |  |  |  |  |  |  |  | **X** |
| *Nodogeneria pseudoscripta* | 50 | 2,000 |  |  |  |  |  |  |  | **X** |  |  |  |  |  |  |  |  |  |  |  |  |  |  |  |  |
| *Nodosaria paupereule* | 50 | 4,000 |  |  |  |  |  | **X** |  |  |  |  |  |  |  |  |  |  |  |  |  |  |  |  |  |  |
| *Nodosaria* sp*.* | 50 | 4,000 |  | **X** |  |  | **X** |  |  |  |  | **X** | **X** | **X** |  |  |  |  |  |  |  |  |  |  |  |  |
| *Nothia excelsa* | 300 | 4,000 |  |  |  |  |  |  |  |  |  | **X** |  |  | **X** | **X** |  | **X** |  |  | **X** |  |  |  |  |  |
| *Osangularia cordieriana* | 600 | 4,000 |  |  |  |  |  |  |  |  |  |  |  |  |  |  |  |  |  |  | **X** | **X** | **X** | **X** |  | **X** |
| *Osangularia p.* | 600 | 4,000 |  |  |  |  |  |  |  |  |  |  |  |  |  |  |  |  |  |  |  |  |  |  |  | **X** |
| *Paratrochamminoides deflexiformis* | 200 | 4,000 |  |  |  |  |  |  |  |  |  |  |  | **X** |  |  |  |  |  |  | **X** |  |  | **X** |  |  |
| *Placentammina placenta* | 200 | 2,000 |  | **X** |  |  |  |  |  |  | **X** |  |  |  | **X** |  |  |  |  | **X** |  |  |  |  |  |  |
| *Pleurostomella* sp*.* | 500 | 2,500 |  | **X** |  |  | **X** |  |  |  |  |  |  | **X** |  |  | **X** |  |  |  |  |  |  |  | **X** | **X** |
| *Pleurostomella wadow* | 500 | 2,500 |  |  | **X** | **X** | **X** | **X** |  |  |  |  |  | **X** |  | **X** | **X** | **X** |  |  | **X** | **X** | **X** |  |  |  |
| *Praebulimina* sp*.* | 500 | 1,500 |  |  | **X** |  |  |  |  |  |  |  | **X** |  |  |  |  |  |  |  |  |  |  |  |  |  |
| *Pullenia* sp*.* | 200 | 4,000 |  |  |  |  |  |  |  |  | **X** |  |  |  |  |  |  |  |  |  | **X** |  |  |  |  |  |
| *Ramulina* sp*.* | 200 | 1,500 |  |  |  |  | **X** |  |  |  |  |  |  |  |  |  |  |  |  |  |  |  | **X** |  |  |  |
| *Ramulina wrightyi* | 200 | 1,500 |  |  |  | **X** |  |  |  |  |  |  |  |  |  |  |  |  |  |  |  |  |  |  |  |  |
| *Reussella* sp*.* 1 | 200 | 3,000 |  | **X** | **X** |  | **X** | **X** | **X** | **X** | **X** |  | **X** | **X** | **X** |  | **X** | **X** | **X** | **X** |  |  |  |  |  | **X** |
| *Reussella szajnochae* | 200 | 3,000 | **X** | **X** | **X** | **X** | **X** | **X** | **X** | **X** | **X** |  |  | **X** |  | **X** |  |  |  | **X** | **X** | **X** |  |  |  |  |
| Sp*iroplectinella dentata* | 400 | 2,500 |  | **X** | **X** | **X** | **X** | **X** | **X** | **X** | **X** | **X** |  | **X** | **X** | **X** | **X** |  |  |  |  |  |  |  | **X** | **X** |
| Sp*iroplectinella* sp*.*1 | 400 | 4,000 |  |  |  |  |  |  | **X** | **X** | **X** | **X** |  |  |  |  |  |  |  |  |  |  |  | **X** | **X** |  |
| *Stensioina pommerana* | 200 | 1,500 | **X** | **X** | **X** |  |  | **X** |  |  |  |  |  | **X** |  |  |  |  |  |  |  |  | **X** |  |  |  |
| *Stilostomella alexanderi* | 500 | 2,000 |  |  |  |  |  |  |  |  |  | **X** |  |  |  |  |  |  |  |  |  |  |  |  |  |  |
| *Stilostomella* sp*.* | 500 | 2,000 |  |  | **X** |  |  |  |  |  | **X** |  |  |  |  |  |  |  |  |  |  |  |  |  | **X** |  |
| *Textularia* sp*.* | 1,500 | 3,500 |  |  | **X** |  |  |  |  |  |  |  |  |  |  |  |  |  |  |  |  |  |  |  |  |  |
| *Thalmannammina* sp*.* | 200 | 3,000 |  |  |  |  |  |  | **X** |  |  |  |  |  |  |  |  |  |  |  |  |  |  |  |  |  |
| *Tritaxia* sp.1 *(tripleura* ?*)* | 500 | 3,000 | **X** |  | **X** |  |  |  |  | **X** |  | **X** |  | **X** | **X** |  | **X** |  | **X** |  |  |  |  |  | **X** | **X** |
| *Tritaxia eggeri* | 500 | 2,700 |  |  |  |  |  |  |  | **X** |  |  |  |  |  |  |  | **X** | **X** | **X** |  | **X** | **X** | **X** | **X** | **X** |
| *Trochammina boehmi* | 1,600 | 4,000 |  |  |  |  |  |  |  |  |  |  |  |  |  |  |  |  |  |  |  |  |  |  | **X** |  |
| *Trochammina* sp*.* | 1,600 | 4,000 |  |  |  |  | **X** |  |  |  |  |  |  |  | **X** |  |  |  |  |  | **X** |  |  |  |  |  |
| *Trochamminoides proteus* | 1,600 | 4,000 |  |  |  |  |  |  | **X** |  |  |  |  |  |  | **X** |  |  |  |  |  |  |  |  |  |  |
| *Verneulina muensteri* | 500 | 1,500 |  |  |  |  |  |  |  |  |  |  | **X** |  |  |  |  |  |  |  |  |  |  |  |  |  |
| *Verneulina* sp*.* | 500 | 1,500 |  | **X** |  |  |  |  |  |  | **X** |  |  |  |  |  | **X** |  |  |  |  |  |  |  |  |  |
